# Pericapsular Nerve Group Block Combined With Lateral Femoral Cutaneous Nerve Block Versus Fascia Iliaca Compartment Block for Hip Surgery: A Systematic Review and Meta-Analysis

**DOI:** 10.7759/cureus.114063

**Published:** 2026-08-06

**Authors:** Abdullah M Alharran, Mahnd Alaenezi, Fahad AlAbduljader, Abdulwahab Althuwaini, Mohammad AlMutairi, Afaq Alkhalil, Salah Termos

**Affiliations:** 1 College of Medicine &amp; Medical Sciences, Arabian Gulf University, Manama, BHR; 2 Department of General Surgery, Al-Amiri Hospital, Kuwait City, KWT; 3 Department of General Surgery, American University of Beirut Medical Center, Beirut, LBN

**Keywords:** anesthesia, hip fracture, motor sparing, postoperative analgesia, total hip arthroplasty

## Abstract

Postoperative pain after hip surgery remains a critical challenge, and traditional regional blocks like the fascia iliaca compartment block (FICB) frequently cause quadriceps motor weakness. The pericapsular nerve group (PENG) block, combined with a lateral femoral cutaneous nerve (LFCN) block, is a novel motor-sparing alternative. This systematic review and meta-analysis compares the efficacy and safety of PENG + LFCN versus FICB in patients undergoing hip surgery. A systematic search was conducted across five databases (PubMed, Scopus, CENTRAL, Web of Science [WoS], and Google Scholar) up to December 2025. Eligible randomized controlled trials (RCTs) were assessed for methodological quality using the RoB-2 tool. Primary outcomes were pain scores and quadriceps motor strength (QMS). Secondary outcomes included rescue analgesia, time to first ambulation, and complications. Standardized mean differences (SMDs) and risk ratios (RRs) were pooled with 95% confidence interval (CI). Five RCTs involving 382 patients were included. The PENG + LFCN block significantly improved QMS compared to FICB at 6 hours (SMD: 1.04, 95% CI [0.63, 1.45]; p < 0.001), 12 hours (SMD: 2.02, 95% CI [0.64, 3.40]; p < 0.001), and 24 hours (SMD: 0.90, 95% CI [0.12, 1.68]; p = 0.02). Furthermore, the combined block significantly decreased pain scores at rest at 12 hours (SMD: -0.88, 95% CI [-1.62, -0.15]; p = 0.02), 24 hours (SMD: -0.80, 95% CI [-1.41, -0.19]; p = 0.01), and 48 hours (SMD: -1.04, 95% CI [-1.66, -0.42]; p < 0.001). The PENG + LFCN block was associated with a significant reduction in rescue analgesia requirements (RR: 0.44, 95% CI [0.22, 0.86]; p = 0.02) and time to first ambulation (MD: -4.08 hours, 95% CI [-7.37, -0.80]; p = 0.01). The PENG + LFCN block was associated with a significant motor-sparing effect and reduced pain scores at rest compared to FICB, resulting in faster ambulation and lower requirements for rescue analgesia. Accordingly, this combined block may represent a better alternative for optimal postoperative recovery in hip surgery, but more trials remain warranted.

## Introduction and background

Globally, the frequency of hip surgeries, including hip fracture repairs, total hip arthroplasty (THA), and hip arthroscopy, has been increasing significantly due to an aging population and longer life expectancy [1]. Still, severe postoperative pain remains a critical challenge often requiring high doses of opioids [2], which can lead to several adverse events, such as nausea, sedation, and delirium [3,4]. As a result, Enhanced Recovery After Surgery (ERAS) protocols have instituted a novel "gold standard" of care that highlights opioid-sparing multimodal analgesia [5]. Early ambulation is a primary objective of ERAS protocols, which is essential to reduce the incidence of life-threatening complications, including deep vein thrombosis, pulmonary embolism, and pneumonia [6].

Currently, the fascia iliaca compartment block (FICB) is a widely accepted regional anesthesia technique for hip surgery [7]; however, it has significant limitations. FICB involves a volume-dependent spread to block the femoral nerve, thus causing severe quadriceps motor weakness [8]. This motor impairment is clinically significant because it increases the risk of iatrogenic falls, impeding patient involvement in early rehabilitation [9]. Additionally, the FICB's ability to cover the obturator nerve is inconsistent, which can result in insufficient analgesia to the medial hip capsule [10]. This led to an increasing trend in investigating novel nerve block interventions to overcome these limitations.

Accordingly, the pericapsular nerve group (PENG) block was introduced as a novel, motor-sparing alternative [11]. By selectively targeting the articular sensory branches of the femoral, obturator, and accessory obturator nerves, the PENG block provides adequate joint analgesia while preserving quadriceps muscle strength (QMS) [12]. However, the PENG block does not provide anesthesia to the skin of the lateral thigh, which is the primary incision site for many hip procedures [12]. Therefore, the combination of the PENG block with a lateral femoral cutaneous nerve (LFCN) block has been proposed to provide effective deep joint analgesia via the PENG block and superficial incision analgesia via the LFCN, while preserving motor function [13].

Still, the clinical evidence on the combined PEGN + LFCN block remains inconsistent. Recently, some randomized controlled trials (RCTs) have compared this combination with FICB, yielding conflicting results [14-18]. Some trials reported superior analgesia and motor sparing with the PENG + LFCN combination [15,18], while others found no significant difference in pain scores or functional outcomes [14,16]. Thus, there is no consensus on whether the combined block is clinically better than the current FICB. This systematic review and meta-analysis aims to synthesize current evidence comparing the analgesic efficacy, motor-sparing effects, and safety of the PENG + LFCN block versus FICB in patients undergoing hip surgery.

## Review

Methods and materials

Review Protocol and Reporting Style

The protocol for this systematic review was prospectively registered in the PROSPERO database (registration ID: CRD420261285038). The conduct and reporting of the study followed the Preferred Reporting Items for Systematic Reviews and Meta-Analyses (PRISMA) guidelines [19] and complied with the methodological standards described in the Cochrane Handbook for Systematic Reviews of Interventions [20]. Ethical approval was not required because the analysis was based exclusively on data from previously published studies.

Information Sources and Search Strategy

A comprehensive systematic literature search was executed on December 6, 2025, across five electronic databases: PubMed, Scopus, the Cochrane Central Register of Controlled Trials (CENTRAL), Google Scholar, and Web of Science (WoS). The search strategy employed a Boolean combination of Medical Subject Headings (MeSH) keywords to form the following search strategy: “(“Pericapsular nerve group” OR PENG OR “PENG block” OR PNGB) AND (“Lateral femoral cutaneous nerve” OR LFCN OR “LFCN block”) AND (“Fascia iliaca” OR FICB OR FIB OR S-FICB OR “Fascia iliaca compartment block”) AND (Hip AND (Arthroplasty OR Replacement OR Fracture* OR Arthroscopy OR Proximal femoral OR Proximal femur)).” No restrictions were imposed regarding language, country of origin, or publication status.

To enhance the completeness of the search, the reference lists of all included studies were manually reviewed, and trial registries, including ClinicalTrials.gov and the World Health Organization (WHO) International Clinical Trials Registry Platform (ICTRP), were searched. Additional sources, such as ResearchGate, were also examined to identify potentially eligible unpublished studies. When required, corresponding authors were contacted to obtain missing information or to clarify study details.

Eligibility Criteria

Study inclusion was restricted to RCTs satisfying the following Population, Intervention, Control, and Outcome (PICO) framework:

Population: Adult patients (age≥ 18 years) undergoing hip surgery (including hip fracture repair, elective THA, and hip arthroscopy).

Intervention: PENG block combined with LFCN block, irrespective of the dosing regimen of local anesthetics.

Control: FICB, including standard infra-inguinal FICB and supra-inguinal FICB, irrespective of the dosing regimen of local anesthetics.

Outcomes: the primary endpoints were pain scores at rest and during movement, as well as QMS. Secondary endpoints included rescue analgesia, opioid consumption, block duration, time to first ambulation, and complications (i.e., post-operative nausea and vomiting (PONV), pruritus, infection, and hematoma).

Study Selection Process

Records were managed and screened using an electronic platform. Following automated de-duplication, two investigators independently screened articles in a two-stage process: first by title and abstract, followed by a full-text review of potentially eligible studies. Any discrepancies regarding study inclusion were resolved through consensus-based discussion.

Data Extraction and Review Items

Data extraction was conducted independently by two reviewers using a pilot-tested Microsoft Excel proforma (Microsoft Corp., Redmond, WA). A senior author resolved inconsistencies. The data extraction form captured three primary sections:

Study characteristics: study ID, location, design, sample size, PENG + LFCN details, FICB details, surgery type, anesthesia drugs, adjuvant analgesia, primary outcome, pain assessment score, main inclusion criteria, and follow-up duration.

Participant data: sample distribution, age, gender, American Society of Anesthesiologists (ASA) classification, and body mass index (BMI).

Outcome data: pain scores at rest and during movement, QMS, rescue analgesia, opioid consumption, block duration, time to first ambulation, and complications (i.e., PONV, pruritus, infection, and hematoma).

Dichotomous outcomes were extracted as event counts and total sample sizes, while continuous outcomes were collected as means and standard deviations. When studies reported data as medians with interquartile ranges, these were converted to means and standard deviations using the methods described by Wan et al. [21].

Outcomes Assessment Tools

Postoperative pain was evaluated using either the visual analog scale (VAS) or the numerical rating scale (NRS), both of which ranged from 0 (no pain) to 10 (worst imaginable pain). For the meta-analysis of pain scores, a lower mean value was defined as the direction of a positive effect, indicating superior analgesia. Also, QMS was assessed using manual muscle testing grading systems, such as the Medical Research Council (MRC) scale [22], with a higher score representing greater muscle strength.

Risk of Bias and Certainty of Evidence Assessment

The methodological quality of the included RCTs was evaluated using the revised Cochrane Risk of Bias tool (RoB-2) [23]. Two reviewers independently assessed each study across five domains: the randomization process, deviations from intended interventions, missing outcome data, outcome measurement, and selection of the reported results. Any disagreements were resolved through discussion. In addition, the certainty of evidence was assessed using the Grading of Recommendations Assessment, Development, and Evaluation (GRADE) framework, which considers risk of bias, inconsistency, indirectness, imprecision, and publication bias [24,25].

Meta-Analysis and Heterogeneity Assessment

Statistical analysis was performed using Stata/SE version 19.5 (Stata Corp., College Station, TX). Continuous outcomes were analyzed using Hedges' g, a standardized mean difference (SMD), for variables with different measurement scales (pain scores, QMS), while the mean difference (MD) was utilized for variables with uniform units. Dichotomous outcomes were analyzed using risk ratios (RRs). An REML (restricted maximum likelihood) random-effects model was adopted if significant statistical or clinical heterogeneity was detected. Heterogeneity was evaluated using the chi-squared (χ²) test and the I² statistic. A p-value less than 0.1 for the χ² test or an I² value of 50% or higher indicated significant heterogeneity. In cases of significant heterogeneity, robustness was assessed via leave-one-out sensitivity analysis. To further investigate sources of heterogeneity, we employed Galbraith plots, which graph the standardized effect size against precision. Studies falling outside the 95% confidence bounds were identified as significant contributors to heterogeneity. Publication bias assessment was not feasible, as all analyzed outcomes included fewer than 10 studies [26]. Finally, to assess the conclusiveness of the cumulative evidence and control for type I errors associated with repeated significance testing in meta-analyses, we conducted trial sequential analysis (TSA) [27]. The required information size (RIS), analogous to the sample size in a single RCT, was estimated based on a two-sided alpha of 0.05, 80% statistical power, and an assumed relative risk reduction (RRR) of 20%.

Results

Systematic Literature Search and Study Selection

**Figure 1:**
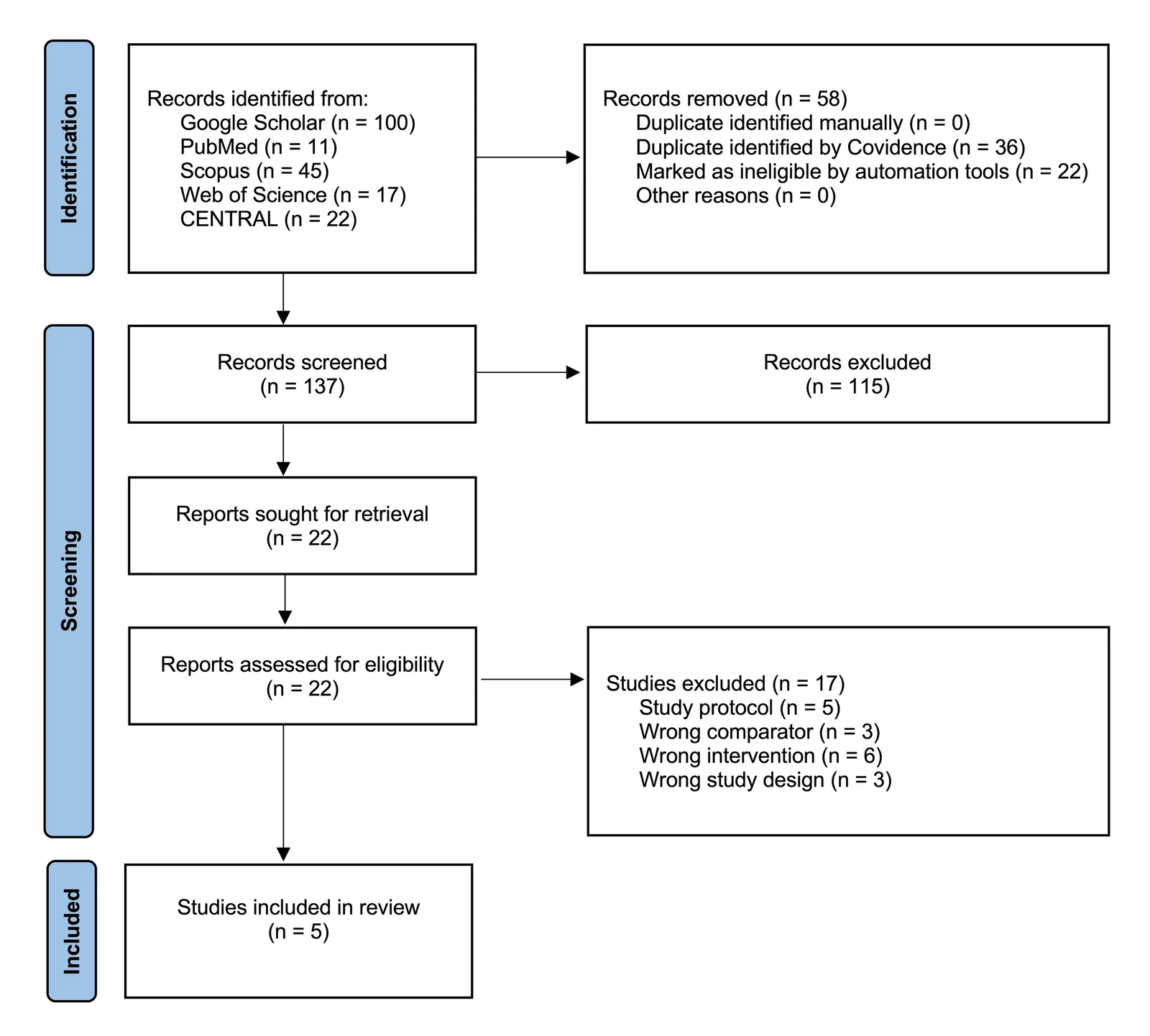
The PRISMA flow diagram illustrating the systematic study selection process. Studies included: [14-18] PRISMA, Preferred Reporting Items for Systematic Reviews and Meta-Analyses

A total of 195 records were identified through database searching. After removing 58 duplicate or ineligible records, 137 records remained for screening. During the screening process, 115 records were excluded. Then, 22 full texts were assessed for eligibility. Of these, 17 studies were excluded for different reasons. Finally, five trials [14-18] met the inclusion criteria and were included in the systematic review (Figure 1).

Characteristics of the Included Studies and Participants

This review synthesized five RCTs and 382 patients [14-18]. Two studies were conducted in China, and one each in India, Egypt, and Italy. The intervention consisted of a combined ultrasound-guided PENG and LFCN block; still, the local anesthetic regimen differed between trials. Local anesthetic concentration ranged from 0.25% bupivacaine to 0.5% ropivacaine, with one study [17] also including the adjuvant dexamethasone. Further details on the study design of the included trials are outlined in Table 1. Also, details on the included patients’ baseline data are outlined in Table 2.

**Table 1:** Summary of the characteristics of the included randomized controlled trials. AHS, arthroscopic hip surgery; ASA, American Society of Anesthesiologists; DAA, direct anterior approach; IV, intravenous; FICB, fascia iliaca compartment block; LFCN, lateral femoral cutaneous nerve; mg/kg, milligram per kilogram; MRC, Medical Research Council; NRS, numerical rating scale; PCIA, patient-controlled intravenous analgesia; PENG, pericapsular nerve group; q8h, every 8 hours; RCT, randomized controlled trial; THA, total hip arthroplasty; VAS, visual analog scale

Study	Study Design	Country	Total Participants	PENG + LFCN Details	FICB Details	Surgery Type	Anesthesia Drugs	Adjuvant Analgesia	Primary Outcome	Pain Assessment Score	Main Inclusion Criteria	Follow-up Duration
Smruthi et al. 2025 [17]	RCT	India	60	Timing: before spinal anesthesia. PENG: 20 mL 0.25% bupivacaine + 8 mg dexamethasone, LFCN: 5 mL 0.25% bupivacaine	Timing: before spinal anesthesia. FICB: 25 mL 0.25% bupivacaine + 8 mg dexamethasone	Hip fracture surgery (neck of femur, intertrochanteric, subtrochanteric)	Spinal: 2.8 mL 0.5% heavy bupivacaine + 60 µg buprenorphine	IV paracetamol 1 g q8h; Rescue: tramadol 1 mg/kg and/or diclofenac	Duration of analgesia, pain scores, and need for systemic analgesics	NRS (0-10)	Age >18 years, ASA I-II, undergoing surgery for intertrochanteric, subtrochanteric, or neck of femur fractures	48 hours
Liang et al. 2023 [14]	RCT	China	92	Timing: before anesthesia induction. PENG: 20 mL 0.33% ropivacaine, LFCN: 10 ml 0.33% ropivacaine	Timing: before anesthesia induction, S-FICB: 30 mL 0.33% ropivacaine	THA	General: propofol, sufentanil, vecuronium, sevoflurane, remifentanil	IV dexamethasone 10 mg, PCIA (tramadol + flurbiprofen axetil), IV parecoxib 40 mg/24 h, rescue: oral tramadol	Time to first postoperative walk	VAS (0-10)	Age ≥ 18 years, ASA I-III, elective one-sided THA	48 hours
Liu et al. 2024 [15]	RCT	China	78	Timing: before anesthesia induction. PENG: 35 mL 0.375% ropivacaine, LFCN: 5 mL 0.375% ropivacaine	Timing: before anesthesia induction, FICB: 40 mL 0.375% ropivacaine	Hip arthroscopy	General: midazolam, propofol, sufentanil, cisatracurium, remifentanil	PCIA (sufentanil + palonosetron); oral celecoxib 200 mg q12h; rescue: IV ketorolac tromethamine	Rate of quadriceps weakness after block, muscle strength grading, and pain score	VAS (0-10)	ASA I-II, Age ≥ 18 years, undergoing AHS	48 hours
Shalaby et al. 2022 [16]	RCT	Egypt	94	Timing: before spinal anesthesia. PENG: 25 ml 0.25% bupivacaine, LFCN: 5 mL 0.25% bupivacaine	Timing: before spinal anesthesia, S-FICB: 30 mL 0.25% bupivacaine	Proximal femoral fracture surgeries	Spinal: 3 mL bupivacaine 0.5% + 20 µg fentanyl	IM Diclofenac sodium 75 mg/12h (if VAS ≥4); Rescue: IV Morphine	Time to perform spinal anesthesia	VAS (0-10)	Age 50-90 years, ASA I-III, elective and emergent proximal femoral fracture surgery	24 hours
Vetrone et al. 2025 [18]	RFC	Italy	58	Timing: immediately after neuraxial anesthesia. PENG: 20 mL 0.5% ropivacaine, LFCN: 10 mL 0.5% ropivacaine	Timing: immediately after neuraxial anesthesia, FICB: 20 mL 0.5% ropivacaine	THA	Spinal: 0.5% hyperbaric bupivacaine + 5 mcg sufentanil	Acetaminophen 1g + Ibuprofen 400 mg q8h; IV Dexamethasone 8 mg + Magnesium 2 g; Rescue: Oral Oxycodone	Quadriceps weakness at 6 h post-block (MRC scale)	NRS (0-10)	Age ≥ 18 years, elective THA via DAA, non-traumatic hip disease	48 hours

**Table 2:** Baseline characteristics of the participants in the included studies. ASA, American Society of Anesthesiologists; BMI, body mass index; FICB, fascia iliaca compartment block; LFCN, lateral femoral cutaneous nerve; PENG, pericapsular nerve group; NR, not reported; SD, standard deviation

Study	Number of Participants in Each Group		Age (Years), Mean (SD)		Gender (Male/Female)		ASA I/II/III/IV		BMI, Mean (SD)	
	PENG + LFCN	FICB	PENG + LFCN	FICB	PENG + LFCN	FICB	PENG + LFCN	FICB	PENG + LFCN	FICB
Smruthi et al., 2025 [17]	30	30	74.00 (NR)	73.70 (NR)	8/22	4/26	ASA I: 3; ASA II: 27	ASA I: 4; ASA II: 26	NR	NR
Liang et al., 2023 [14]	46	46	66.7 (14.4)	67.3 (10.2)	18/28	19/26	ASA II: 26; ASA III: 20	ASA II: 24; ASA III: 21	23.3 (3.7)	24.3 (3.6)
Liu et al., 2024 [15]	39	39	44.1 (13.7)	39.3 (17.7)	22/17	24/15	ASA I: 20; ASA II: 19	ASA I: 19; ASA II: 20	24.6 (3.7)	23.5 (3.9)
Shalaby et al., 2022 [16]	46	48	NR	NR	NR	NR	NR	NR	NR	NR
Vetrone et al., 2025 [18]	29	29	71 (10)	71 (9)	19/10	14/15	Median: 2 [2; 2]	Median: 2 [2; 2]	27.8 (3.9)	28.8 (5.7)

Risk of Bias and Certainty of Evidence

**Figure 2:**
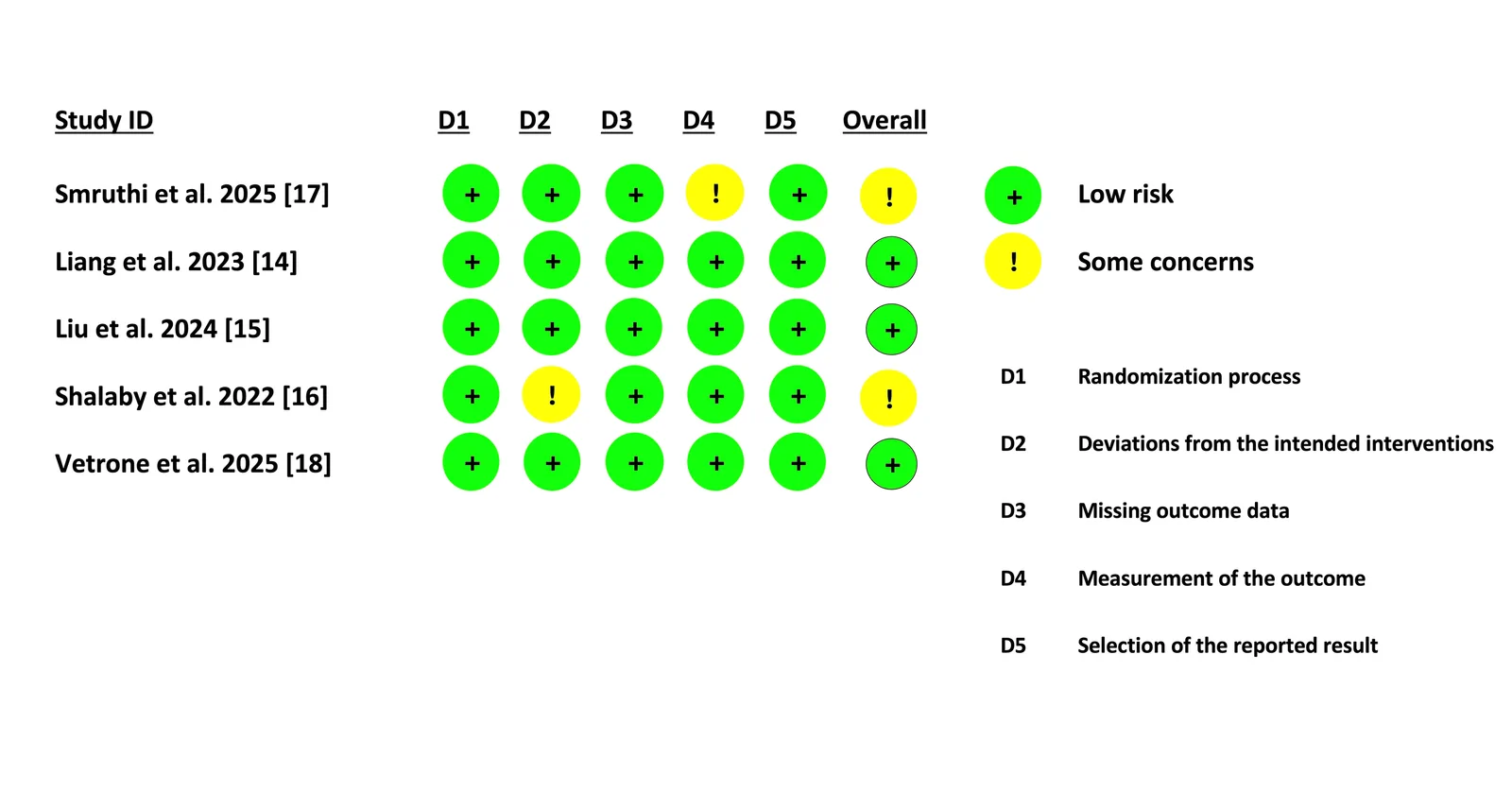
Risk-of-bias graph and summary of the included randomized controlled trials. The panel presents a schematic representation of risks (low = green, some concerns = yellow, and high = red) for specific types of biases of each study in the review [14-18]. Studies: Smruthi et al. [17], Liang et al. [14], Liu et al. [15], Shalaby et al. [16], Vetrone et al. [18]

Three RCTs showed an overall low risk of bias [14,15,18], but two RCTs raised some concerns [16,17] (Figure 2). Smruthi et al. raised concerns about detection bias due to the open-label assessment of subjective outcomes [17]. Additionally, Shalaby et al. raised concerns about performance bias, as they performed a per-protocol analysis rather than an intention-to-treat analysis, and patients were excluded after randomization for "failed block" and "prolonged operation" [16]. Furthermore, the assessment of the certainty of evidence is detailed in Table 3.

**Table 3:** GRADE evidence profile for the assessed outcomes. ^a^Smruthi et al. and Shalaby et al. raised some concerns of bias and had a significant weight in the pooled analysis. ^b^I^2^ > 75%. ^c^There were some differences among the included trials in the intervention details and fascia iliaca compartment block type. ^d^A wide confidence interval. ^e^A low number of participants. ^f^I^2^ > 50%. ^g^A low number of events. Studies: [14-18] Studies: Smruthi et al. [17], Liang et al. [14], Liu et al. [15], Shalaby et al. [16], Vetrone et al. [18] CI, confidence interval; GRADE, Grading of Recommendations Assessment, Development, and Evaluation; MD, mean difference; RR, risk ratio; SMD, standardized mean difference

Certainty assessment							Summary of findings				
Participants (studies) follow-up	Risk of bias	Inconsistency	Indirectness	Imprecision	Publication bias	Overall certainty of evidence	Study event rates (%)		Relative effect (95% CI)	Anticipated absolute effects	
							With FICB	With PENG + LFCN block		Risk with FICB	Risk difference with PENG + LFCN block
Pain at rest (6 hours)											
382 (5 RCTs) [14-18]	Serious^a^	Very serious^b^	Serious^c^	Serious^d^	None	⨁◯◯◯ Very low^a,b,c,d^	-	-	-	-	SMD 0.55 SD lower (1.12 lower to 0.02 higher)
Pain at rest (12 hours)											
232 (3 RCTs) [15-17]	Serious^a^	Very serious^b^	Serious^c^	Very serious^d,e^	None	⨁◯◯◯ Very low^a,b,c,d,e^	-	-	-	-	SMD 0.86 SD lower (1.62 lower to 0.15 lower)
Pain at rest (24 hours)											
382 (5 RCTs) [14-18]	Serious^a^	Very serious^b^	Serious^c^	Serious^d^	None	⨁◯◯◯ Very low^a,b,c,d^	-	-	-	-	SMD 0.8 SD lower (1.41 lower to 0.19 lower)
Pain at rest (48 hours)											
288 (4 RCTs) [14,15, 17,18]	Not serious	Very serious^b^	Serious^c^	Serious^d^	None	⨁◯◯◯ Very low^b,c,d^	-	-	-	-	SMD 1.04 SD lower (1.66 lower to 0.42 lower)
Pain on movement (6 hours)											
322 (4 RCTs) [14-16,18]	Serious^a^	Very serious^b^	Serious^c^	Serious^d^	None	⨁◯◯◯ Very low^a,b,c,d^	-	-	-	-	SMD 0.5 SD lower (0.96 lower to 0.04 lower)
Pain on movement (12 hours)											
172 (2 RCTs) [15,16]	Serious^a^	Not serious	Serious^c^	Very serious^d,e^	None	⨁◯◯◯ Very low^a,c,d,e^	-	-	-	-	SMD 0.38 SD lower (0.72 lower to 0.04 lower)
Pain on movement (24 hours)											
322 (4 RCTs) [14,15, 17,18]	Serious^a^	Not serious	Serious^c^	Not serious	None	⨁⨁◯◯ Low^a,c^	-	-	-	-	SMD 0.17 SD lower (0.4 lower to 0.06 higher)
Pain on movement (48 hours)											
228 (3 RCTs) [14,15,18]	Not serious	Serious^f^	Serious^c^	Very serious^d,e^	None	⨁◯◯◯ Very low^c,d,e,f^	-	-	-	-	SMD 0.45 SD lower (0.92 lower to 0.03 higher)
Quadriceps strength (6 hours)											
288 (4 RCTs) [14,15,17,18]	Not serious	Serious^f^	Serious^c^	Very serious^d,e^	None	⨁◯◯◯ Very low^c,d,e,f^	-	-	-	-	SMD 1.04 SD higher (0.63 higher to 1.45 higher)
Quadriceps strength (12 hours)											
138 (2 RCTs) [15,17]	Serious^a^	Very serious^b^	Serious^c^	Very serious^d,e^	None	⨁◯◯◯ Very low^a,b,c,d,e^	-	-	-	-	SMD 2.02 SD higher (0.64 higher to 3.4 higher)
Quadriceps strength (24 hours)											
288 (4 RCTs) [14,15,17,18]	Not serious	Very serious^a^	Serious^c^	Very serious^d,e^	None	⨁◯◯◯ Very low^a,c,d,e^	-	-	-	-	SMD 0.9 SD higher (0.12 higher to 1.68 higher)
Quadriceps strength (48 hours)											
228 (3 RCTs) [14,15,18]	Not serious	Very serious^a^	Serious^c^	Very serious^d,e^	None	⨁◯◯◯ Very low^a,c,d,e^	-	-	-	-	SMD 0.25 SD higher (0.67 lower to 1.18 higher)
Rescue analgesia requirement											
210 (3 RCTs) [15,17,18]	Serious^a^	Not serious	Serious^c^	Very serious^g^	None	⨁◯◯◯ Very low^a,c,g^	31/105 (29.5%)	13/105 (12.4%)	RR 0.43 (0.25 to 0.74)	31/105 (29.5%)	168 fewer per 1,000 (from 221 fewer to 77 fewer)
Duration of analgesia (hours)											
232 (3 RCTs) [15-17]	Serious^a^	Very serious^a^	Serious^c^	Very serious^d,e^	None	⨁◯◯◯ Very low^a,c,d,e^	-	-	-	117	MD 1.85 hours higher (0.59 lower to 4.29 higher)
Time to first ambulation (hours)											
228 (3 RCTs) [14,15,18]	Not serious	Very serious^a^	Serious^c^	Very serious^d,e^	None	⨁◯◯◯ Very low^a,c,d,e^	-	-	-	114	MD 4.08 hours lower (7.37 lower to 0.8 lower)
Post-operative nausea and vomiting											
382 (5 RCTs) [14-18]	Serious^a^	Not serious	Serious^c^	Very serious^d,g^	None	⨁◯◯◯ Very low^a,c,d,g^	22/192 (11.5%)	17/190 (8.9%)	RR 0.81 (0.46 to 1.40)	22/192 (11.5%)	22 fewer per 1,000 (from 62 fewer to 46 more)

Primary outcomes

Pain Score at Rest

**Figure 3:**
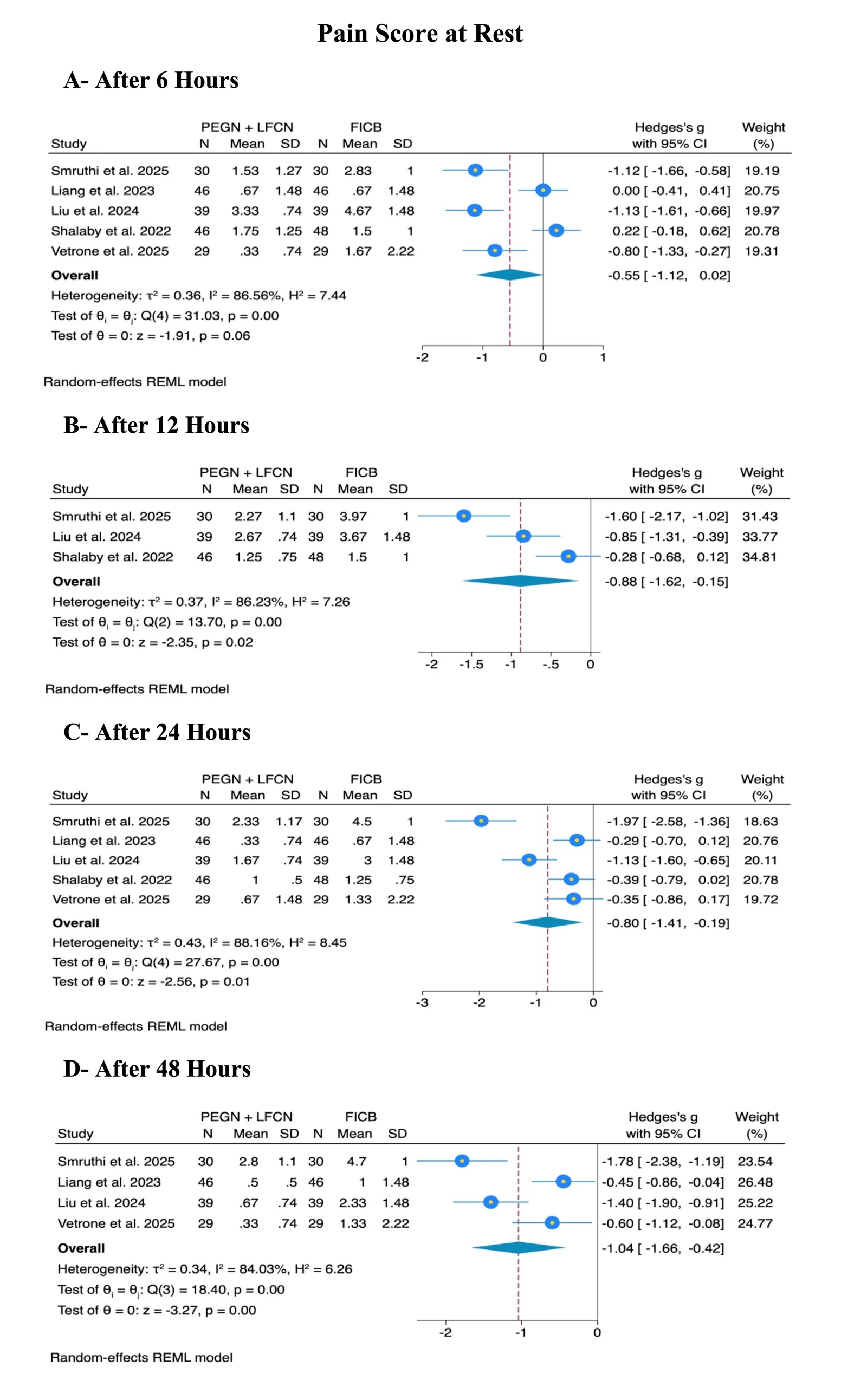
Forest plots of pain at rest. Studies: Smruthi et al. [17], Liang et al. [14], Liu et al. [15], Shalaby et al. [16], Vetrone et al. [18] CI, confidence interval

Compared to FICB, the PENG combined with the LFCN block did not result in a statistically significant reduction in pain scores at rest after 6 hours (SMD: -0.55, 95% CI [-1.12, 0.02], p = 0.06, I² = 86.56%) (Figure 3A). However, pain scores were significantly lower in the PENG + LFCN group after 12 hours (SMD: -0.88, 95% CI [-1.62, -0.15], p = 0.02, I² = 86.23%) (Figure 3B), after 24 hours (SMD: -0.80, 95% CI [-1.41, -0.19], p = 0.01, I² = 88.16%) (Figure 3C), and after 48 hours (SMD: -1.04, 95% CI [-1.66, -0.42], p < 0.001, I² = 84.03%) (Figure 3D).

For the pain score after 6 hours, a leave-one-out sensitivity analysis showed that the result became statistically significant after omitting Liang et al. (p = 0.034) or Shalaby et al. (p = 0.007) (Appendix A). For the pain score after 12 hours, a leave-one-out sensitivity analysis showed that the result became statistically non-significant after omitting Smruthi et al. (p = 0.051) or Liu et al. (p = 0.162) (Appendix B). For the pain score after 24 hours, a leave-one-out sensitivity analysis showed that the result became non-significant after omitting Liu et al. (p = 0.065) (Appendix C). For the pain score after 48 hours, a leave-one-out sensitivity analysis showed that the result remained robustly significant after omitting any of the included studies (Appendix D).

Pain Score During Movement

**Figure 4:**
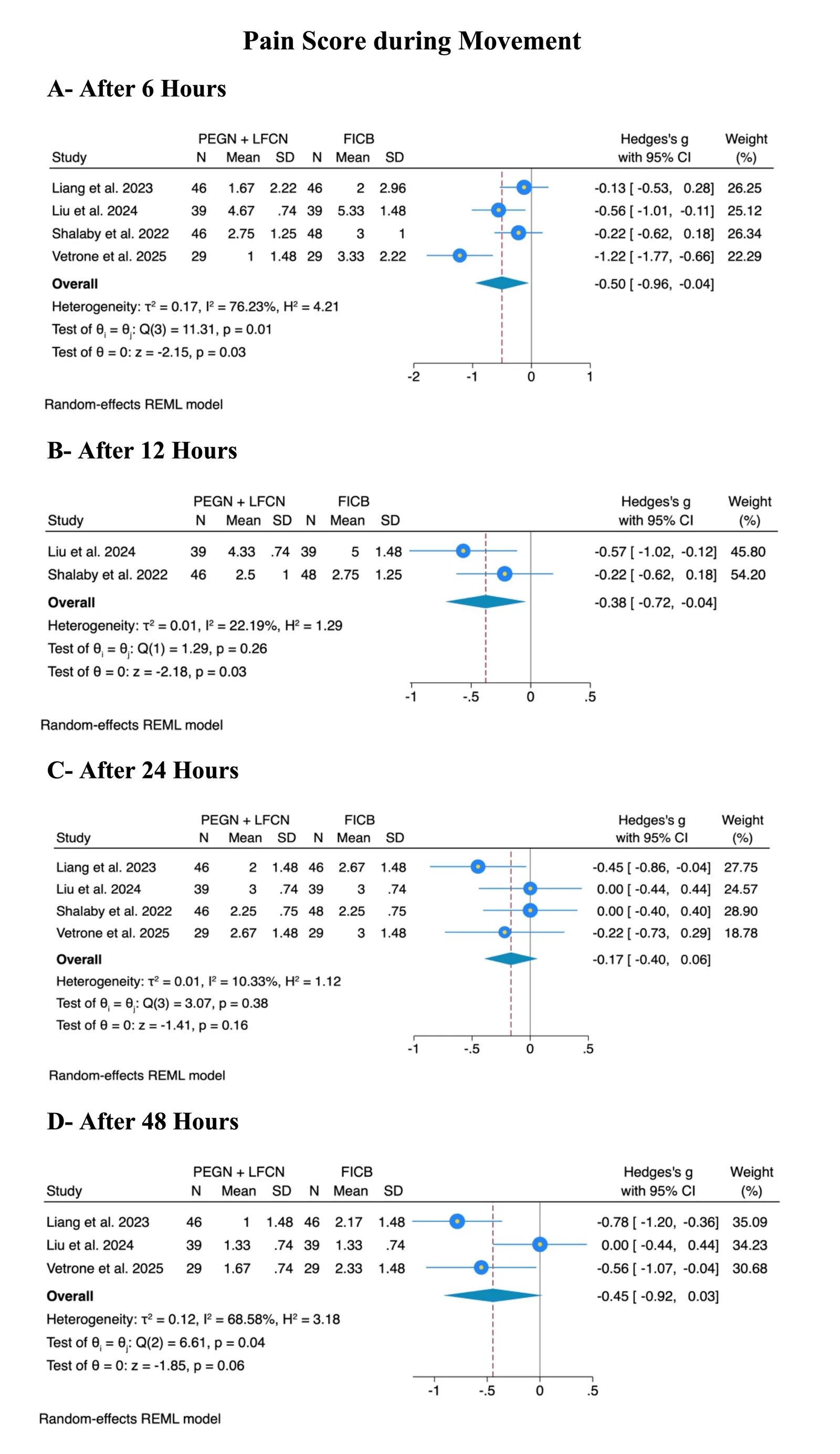
Forest plots of pain during movement Studies: Liang et al. [14], Liu et al. [15], Shalaby et al. [16], Vetrone et al. [18] CI, confidence interval

Compared to FICB, the PENG combined with LFCN block significantly decreased pain scores during movement at 6 hours (SMD: -0.50, 95% CI [-0.96, -0.04], p = 0.03, I² = 76.23%) (Figure 4A) and at 12 hours (SMD: -0.38, 95% CI [-0.72, -0.04], p = 0.03, I² = 22.19%) (Figure 4B). However, this effect faded at 24 hours (SMD: -0.17, 95% CI [-0.40, 0.06], p = 0.16, I² = 10.33%) (Figure 4C) and 48 hours (SMD: -0.45, 95% CI [-0.92, 0.03], p = 0.06, I² = 68.58%) (Figure 4D).

For the pain score at 6 hours, a leave-one-out sensitivity analysis revealed that the result became statistically non-significant after omitting Vetrone et al. (p = 0.021) or Shalaby et al. (p = 0.05) (Appendix E). For the pain score at 48 hours, a leave-one-out sensitivity analysis showed that the result became statistically significant after omitting Liu et al. (p < 0.001) (Appendix F).

Quadriceps Muscle Strength

**Figure 5:**
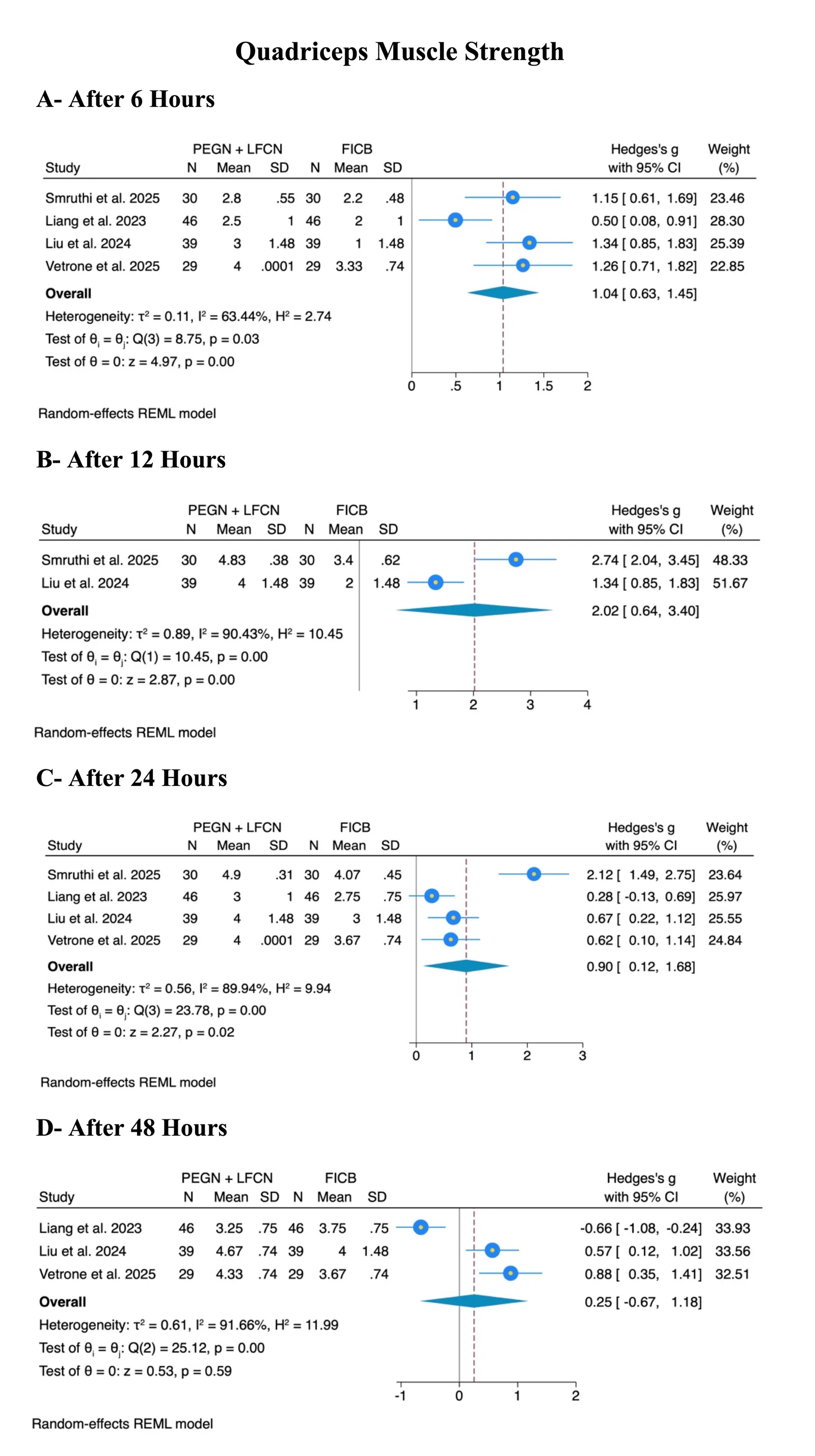
Forest plots of quadriceps muscle strength. Studies: Smruthi et al. [17], Liang et al. [14], Liu et al. [15], Vetrone et al. [18] CI, confidence interval

Compared to FICB, the PENG combined with LFCN block significantly improved QMS at 6 hours (SMD: 1.04, 95% CI [0.63, 1.45], p < 0.001, I² = 63.44%) (Figure 5A), at 12 hours (SMD: 2.02, 95% CI [0.64, 3.40], p < 0.001, I² = 90.43%) (Figure 5B), and at 24 hours (SMD: 0.90, 95% CI [0.12, 1.68], p = 0.02, I² = 89.94%) (Figure 5C). However, there was no statistically significant difference between the groups at 48 hours (SMD: 0.25, 95% CI [-0.67, 1.18], p = 0.59, I² = 91.66%) (Figure 5D).

For QMS at 6 hours, a leave-one-out sensitivity analysis demonstrated that the result remained robustly significant after omitting any individual study (Appendix G). For QMS at 24 hours, the result exhibited considerable instability; a leave-one-out sensitivity analysis revealed that the result became statistically non-significant after omitting either Liu et al. (p = 0.077) or Vetrone et al. (p = 0.069) (Appendix H). For muscle strength at 48 hours, although the primary analysis was non-significant, a leave-one-out sensitivity analysis showed that the result became statistically significant, favoring the PENG + LFCN group after omitting Liang et al. (p < 0.001) (Appendix I), suggesting that this study was a significant source of heterogeneity, obscuring the effect (Appendix J).

Secondary outcomes

Secondary Clinical Outcomes

**Figure 6:**
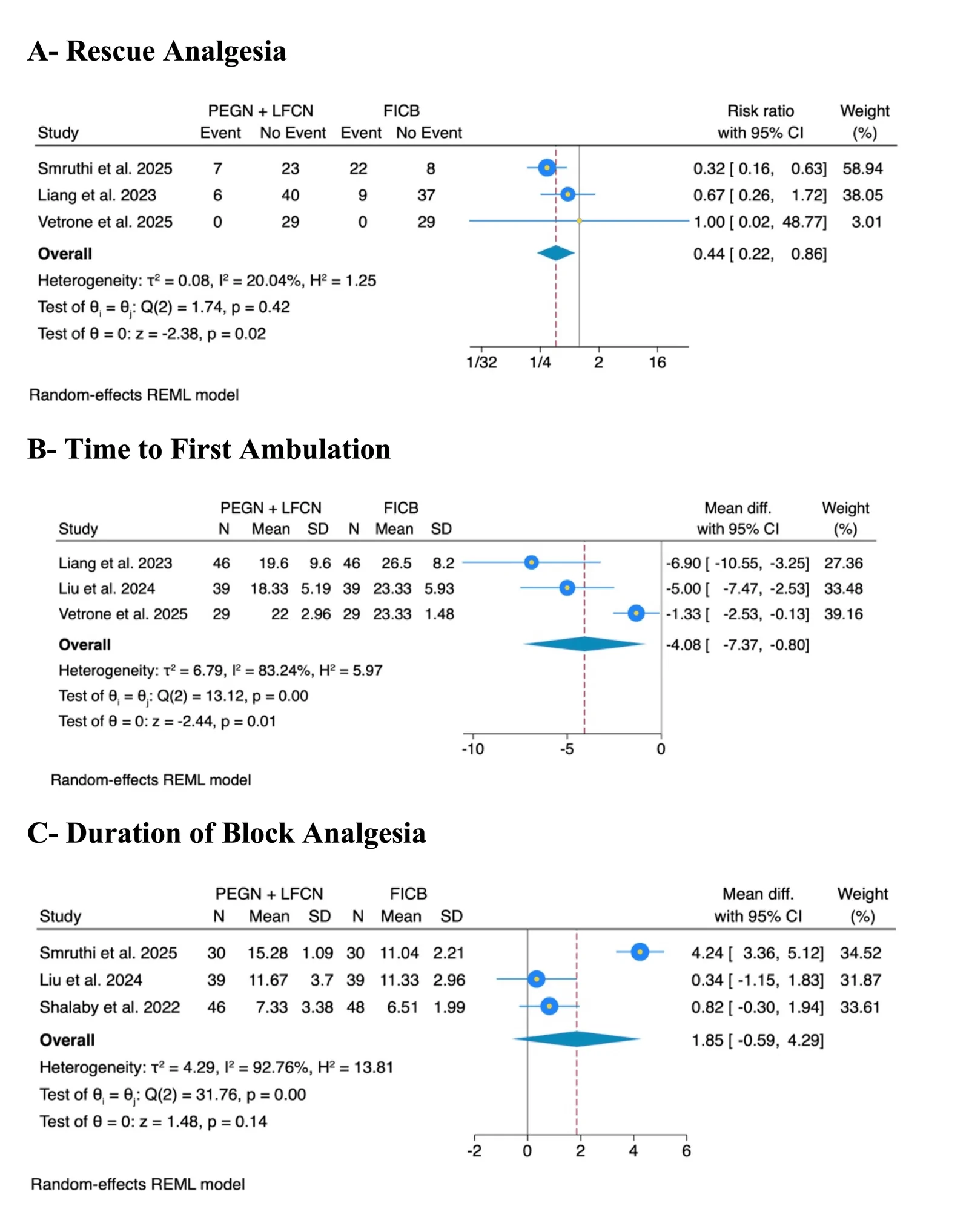
Forest plots of secondary outcomes. Studies: Smruthi et al. [17], Liang et al. [14], Liu et al. [15], Shalaby et al. [16], Vetrone et al. [18] CI, confidence interval

The PENG combined with LFCN block significantly reduced the risk of requiring rescue analgesia compared to the FICB group (RR: 0.44, 95% CI [0.22, 0.86], p = 0.02, I² = 20.04%) (Figure 6A) and decreased the time to first ambulation (MD: -4.08 hours, 95% CI [-7.37, -0.80], p = 0.01, I² = 83.24%) (Figure 6B). However, for the duration of block analgesia, the result did not reach statistical significance (MD: 1.85 hours, 95% CI [-0.59, 4.29], p = 0.14, I² = 92.76%) (Figure 6C).

Analgesia Consumption

We could not pool the analgesia consumption, as the data reporting was heterogeneous among the included trials. Therefore, we provided the following qualitative synthesis: the impact of the PENG block combined with the LFCN block on postoperative analgesic and opioid consumption was evaluated across the included trials, yielding mixed results. Three studies demonstrated a significant reduction in analgesic requirement in the PENG + LFCN group. Specifically, Smruthi et al. reported a significant decrease in total analgesic consumption (11.67 ± 21.50 mg vs. 80 ± 58.13 mg, p < 0.01), compared to the FICB group [17]. Similarly, Vetrone et al. found that total opioid consumption was significantly lower in the combined block group (median 0 vs. 7.5 morphine milligram equivalents, p < 0.001) [18]. In contrast, two studies found no statistically significant differences between the groups. Liang et al. reported comparable intraoperative remifentanil consumption [14], while Shalaby et al. found no significant difference in total morphine consumption during the first 24 hours [16].

Complications

The analysis showed no statistically significant difference between the two groups regarding the incidence of PONV (RR: 0.90, 95% CI [0.51, 1.57], p = 0.71, I² = 0%) (Appendix K) and pruritus (RR: 0.92, 95% CI [0.38, 2.22], p = 0.86, I² = 0%) (Appendix L). Also, none of the included studies reported any instances of infection or hematoma.

Trial Sequential Analysis

Regarding rescue analgesia, the Z-curve crossed the trial sequential monitoring boundary for benefit and the conventional significance boundary, indicating that the current evidence is conclusive and robust, even though the RIS has not been reached (Appendix M). Similarly, for the time to first ambulation, the Z-curve crossed the trial sequential monitoring boundary for benefit, suggesting that the significant reduction in ambulation time is a robust finding and sufficient evidence has been accrued, despite the total sample size not reaching the RIS (Appendix N). For other statistically significant outcomes, we could not perform TSA, as we used SMD as an effect estimate.

Discussion

After synthesizing evidence from five RCTs involving 382 patients, the combined PENG + LFCN block provided a statistically significant higher motor-sparing effect compared to FICB, with preserved QMS at 6, 12, and 24 hours postoperatively. While early resting pain scores (at 6 hours) were comparable between the two interventions, the PENG + LFCN group demonstrated a sustained analgesic benefit, characterized by significantly lower pain scores at rest during the 12-, 24-, and 48-hour intervals. However, there was no significant difference in pain scores during movement. Additionally, the combined block significantly shortened the time to first ambulation and reduced the risk of requiring rescue analgesia.

This motor-sparing effect of the combined PENG + LFCN block offers a promising solution to the FICB block limitation of quadriceps muscle involvement. The PENG block targets the articular sensory branches of the femoral nerve, obturator nerve, and accessory obturator nerve within the myofascial plane between the psoas tendon and the pubic ramus [11]. According to anatomical studies, the anterior hip capsule receives sensory innervation from these high articular branches, whereas the quadriceps muscle's motor branches originate more distally [28,29]. The preservation of QMS at 6, 12, and 24 hours is clinically critical, as this window corresponds to the immediate postoperative period, during which early ambulation is prioritized [30]. Quadriceps weakness has been identified as a risk factor for iatrogenic falls and delayed discharge [31]. By potentially limiting femoral motor involvement, the PENG + LFCN block may facilitate earlier participation in rehabilitation. In contrast, the FICB, especially the supra-inguinal approach, uses a large injection volume, spreading proximally to the lumbar plexus [32]. As a result, this mechanism leads to blocking the main trunk of the femoral nerve, leading to significant quadriceps weakness, which has been reported in up to 70% of patients receiving FICB [33].

Moreover, while the PENG + LFCN block showed better rates of analgesia during rest, the pain scores during movement were similar to those of FICB. This conflict between resting and dynamic pain warrants careful interpretation. The PENG block effectively targets the deep articular pain originating from the highly innervated anterior hip capsule [34]. However, cutaneous innervation of the lateral thigh, the primary incision site for most hip surgeries, is not covered [35]. Adding an LFCN block addresses this gap, creating a comprehensive analgesic block that covers both deep visceral/articular pain (PENG) and superficial somatic pain (LFCN) [14]. This dual-target approach likely explains the significant reduction in rescue analgesia and lower pain scores at 12, 24, and 48 hours compared to FICB. Additionally, while the FICB can block the LFCN, its effect on the obturator nerve is frequently variable (10% to 60%), which may result in insufficient anesthesia of the medial joint capsule [36].

Furthermore, the functional benefits of the PENG + LFCN block extended beyond pain scores. Our meta-analysis showed a significant reduction in the time to first ambulation. This early ambulation is directly linked to the motor-sparing results, as patients are likely to walk sooner, not only because they are in less pain but also because their leg strength is preserved [6]. For ERAS protocols, getting patients moving quickly is a vital measure, which correlates with shorter hospitalizations and fewer complications, such as deep vein thrombosis and pneumonia [6]. Liang et al. specifically noted that patients in the PENG + LFCN group walked approximately 7 hours sooner than those in the S-FICB group [14], a clinically meaningful difference in a fast-track surgery setting.

Additionally, the reduction in rescue analgesia requirements and opioid consumption is a cornerstone of modern multimodal analgesia [37]. Opioids are associated with dose-dependent side effects, including nausea, sedation, urinary retention, and ileus, all of which can delay hospital discharge [38]. Smruthi et al. and Vetrone et al. reported significant reductions in total opioid consumption in the PENG + LFCN group [17,18]. While the difference in PONV was not statistically significant in our pooled analysis, the trend towards needing less rescue analgesia suggests an improved recovery process. The observed improvements in early ambulation and reduced opioid consumption suggest that the PENG + LFCN block may offer significant economic and systemic advantages. Future research is warranted to quantify these benefits, particularly regarding hospital length of stay and overall resource utilization.

To the best of our knowledge, this meta-analysis is the first to investigate the efficacy of PENG + LFCN block versus FICB block in hip surgery. Also, our methods strictly adhered to PRISMA and Cochrane guidelines, pooling only RCTs. Therefore, our results can be considered the latest and comprehensive evidence in this regard. Still, this systematic review is not without limitations. First, significant heterogeneity was observed across several outcomes, which likely stems from some clinical variations among the included trials, including the types of surgery (from hip fractures (trauma) to elective THA and hip arthroscopy). The pain trajectories and inflammatory responses vary significantly between these procedures, with trauma patients usually experiencing a higher level of pain [39]. Second, there were variations in the block techniques, including the volume of local anesthetic (for the PENG block, which ranged from 20 mL to 35 mL [1,3], and for the FICB, which ranged from 20 mL to 40 mL). Anatomical studies suggest that larger volumes in PENG blocks may cause unintentional spread of the femoral nerve, potentially affecting motor-sparing outcomes [40]. Third, the control group included both supra-inguinal and infra-inguinal approaches to FICB, which have different efficacy profiles regarding obturator nerve coverage [41]. Fourth, the measurement tools introduced subjectivity, as muscle strength was assessed using various scales, and pain was measured using VAS or NRS, which are subjective patient-reported outcomes. Also, the MRC scale is ordinal, not truly continuous; thus, pooling QMS as SMD may assume a degree of comparability that may not be fully justified. Additionally, median to mean conversion introduces approximation error. Finally, despite including five RCTs, the total sample size remained relatively small (<400 patients), which limited our ability to conduct subgroup analyses based on the above variations.

Future research should focus on standardizing the PENG block technique, specifically determining the optimal anesthetic volume that provides maximum analgesia without motor spillover. Dose-finding studies are needed to establish whether lower volumes (15-20 mL) are sufficient for analgesia while guaranteeing motor sparing [42]. Moreover, more large-scale RCTs are needed in the hip fracture patients, who would gain the most benefit from motor-preserving, opioid-sparing techniques to prevent delirium and facilitate the immediate mobilization required to reduce complications and mortality.

## Conclusions

The combined PENG + LFCN block may represent a promising motor‑sparing alternative to FICB for selected patients undergoing hip surgery, but current evidence does not yet support routine preferential use. PENG + LFCN block showed statistically significant higher QMS compared to the FICB block and lower static pain scores up to 48 hours. As a result, the PENG + LFCN block significantly decreased the need for rescue analgesia and the time to first ambulation. Still, the current evidence is based on five small RCTs, with some variations, warranting further large-scale trials before full clinical endorsement. Additionally, factors such as hip fracture, THA, and arthroscopy involve different ages, frailty levels, pain trajectories, and anesthetic techniques, and rehabilitation goals may modify the outcome of the procedure type.

## Figures and Tables

**Figure 7:**
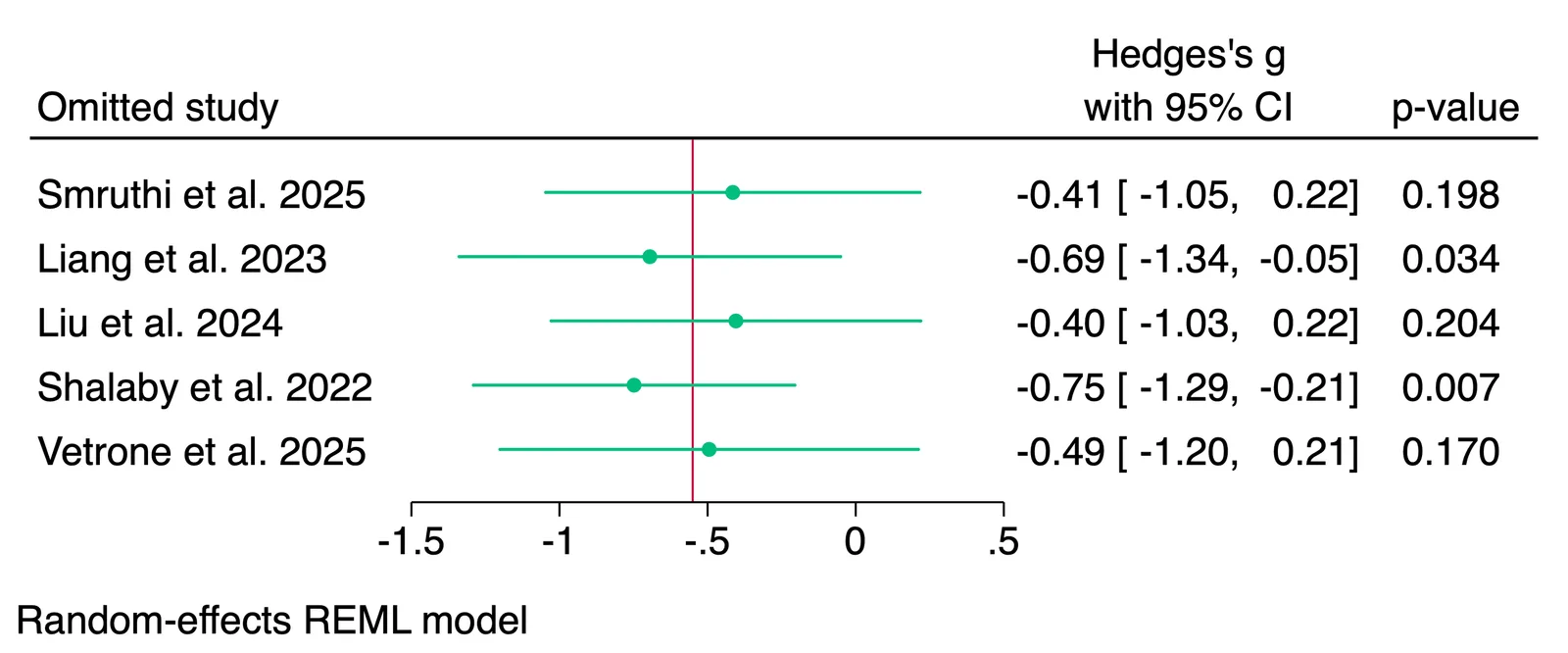
Leave-one-out sensitivity analysis of pain at rest after 6 hours. Studies: Smruthi et al. [17], Liang et al. [14], Liu et al. [15], Shalaby et al. [16], Vetrone et al. [18]

**Figure 8:**
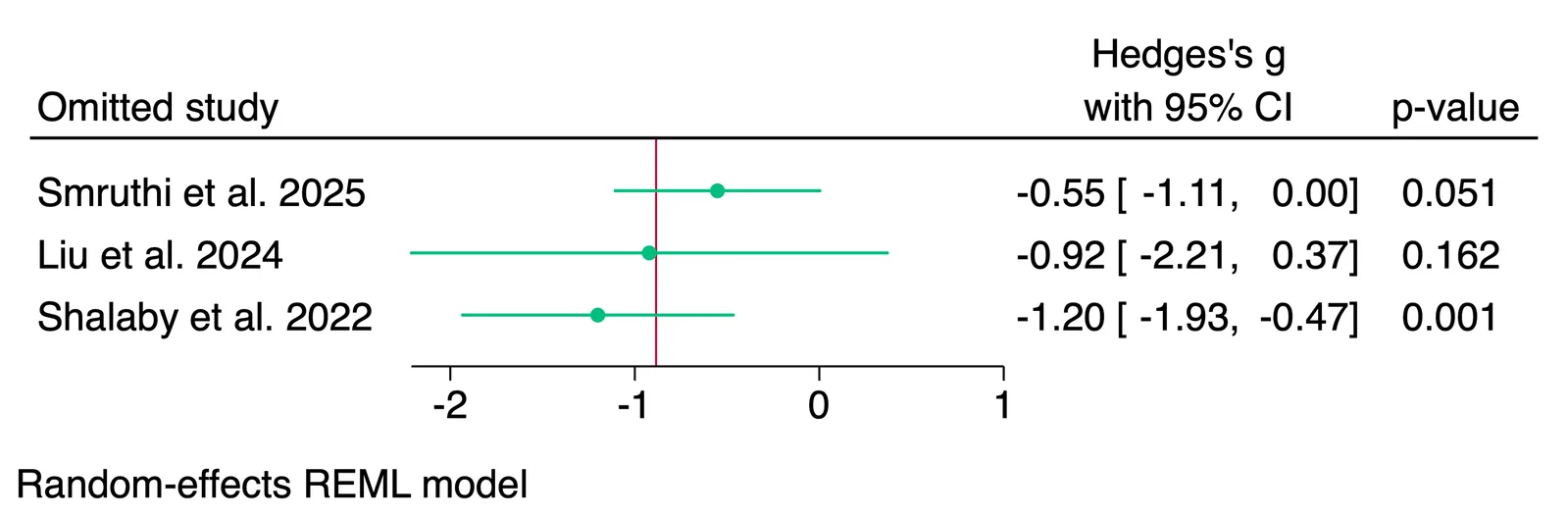
Leave-one-out sensitivity analysis of pain at rest after 12 hours. Studies: Smruthi et al. [17], Liu et al. [15], Shalaby et al. [16]

**Figure 9:**
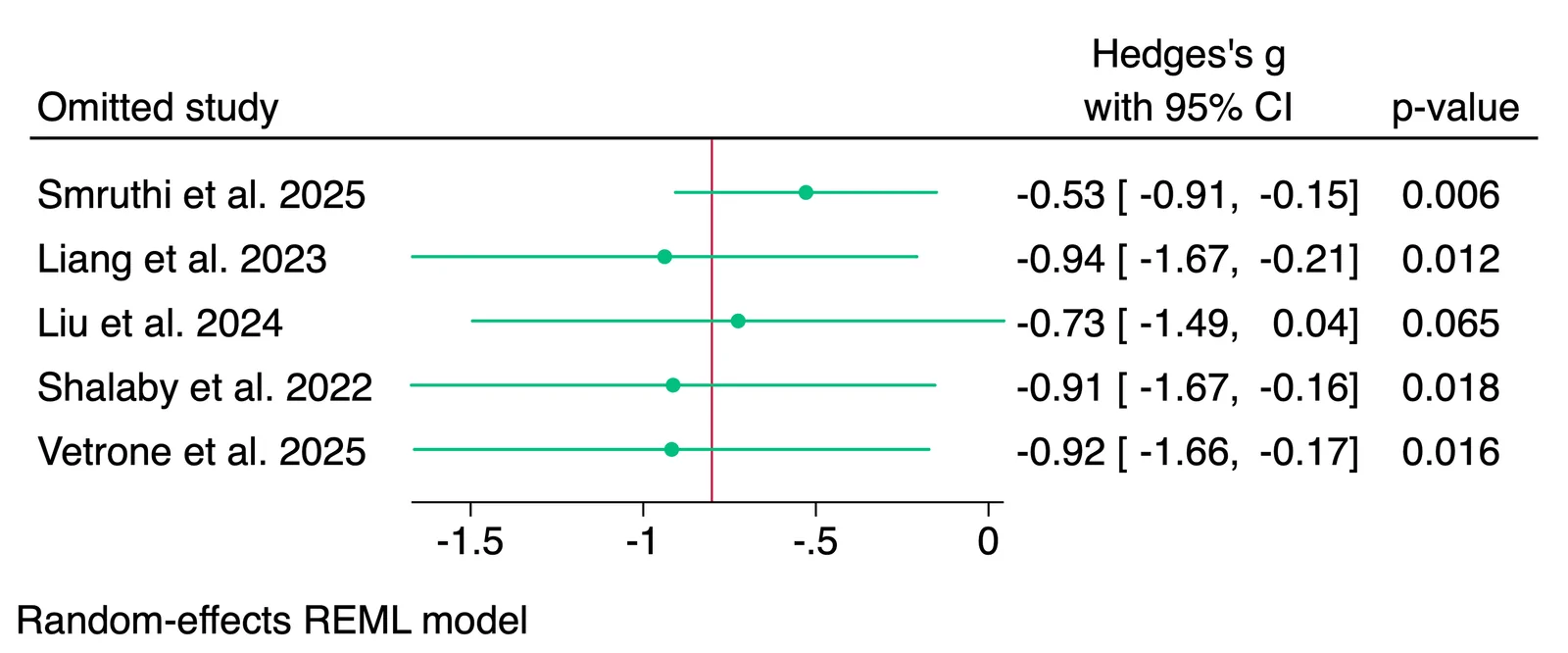
Leave-one-out sensitivity analysis of pain at rest after 24 hours. Studies: Smruthi et al. [17], Liang et al. [14], Liu et al. [15], Shalaby et al. [16], Vetrone et al. [18]

**Figure 10:**
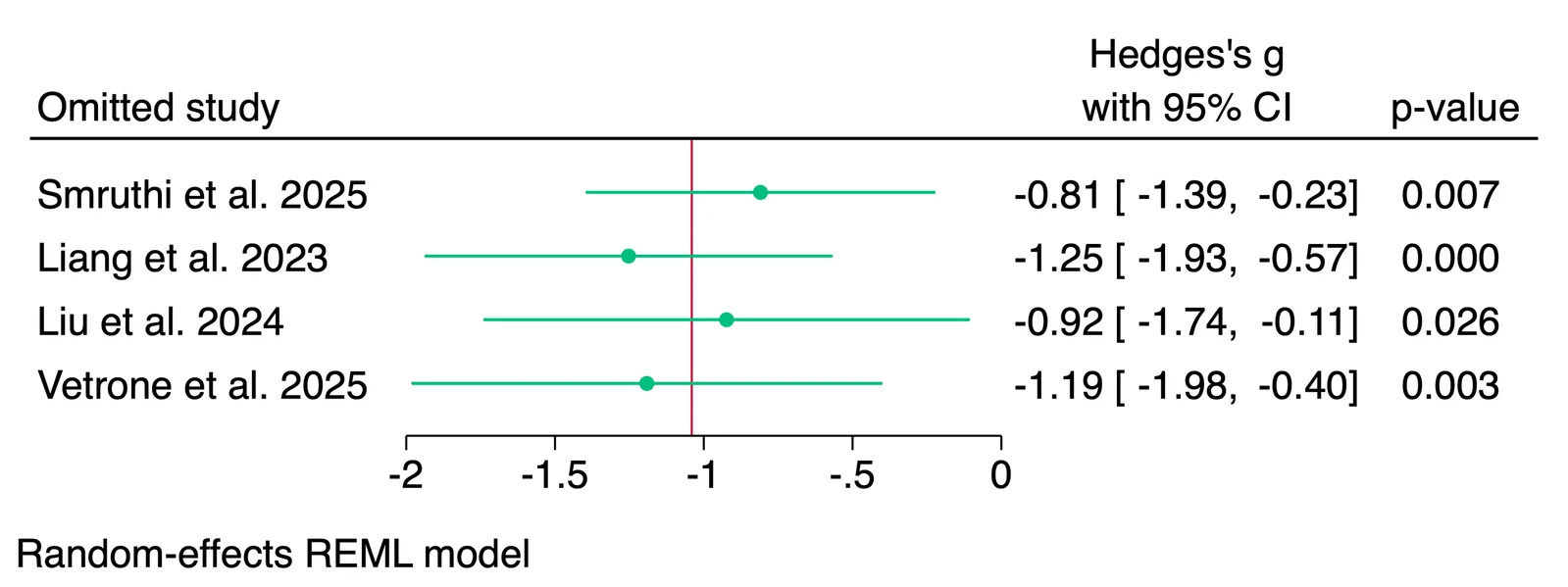
Leave-one-out sensitivity analysis of pain at rest after 48 hours. Studies: Smruthi et al. [17], Liang et al. [14], Liu et al. [15], Vetrone et al. [18]

**Figure 11:**
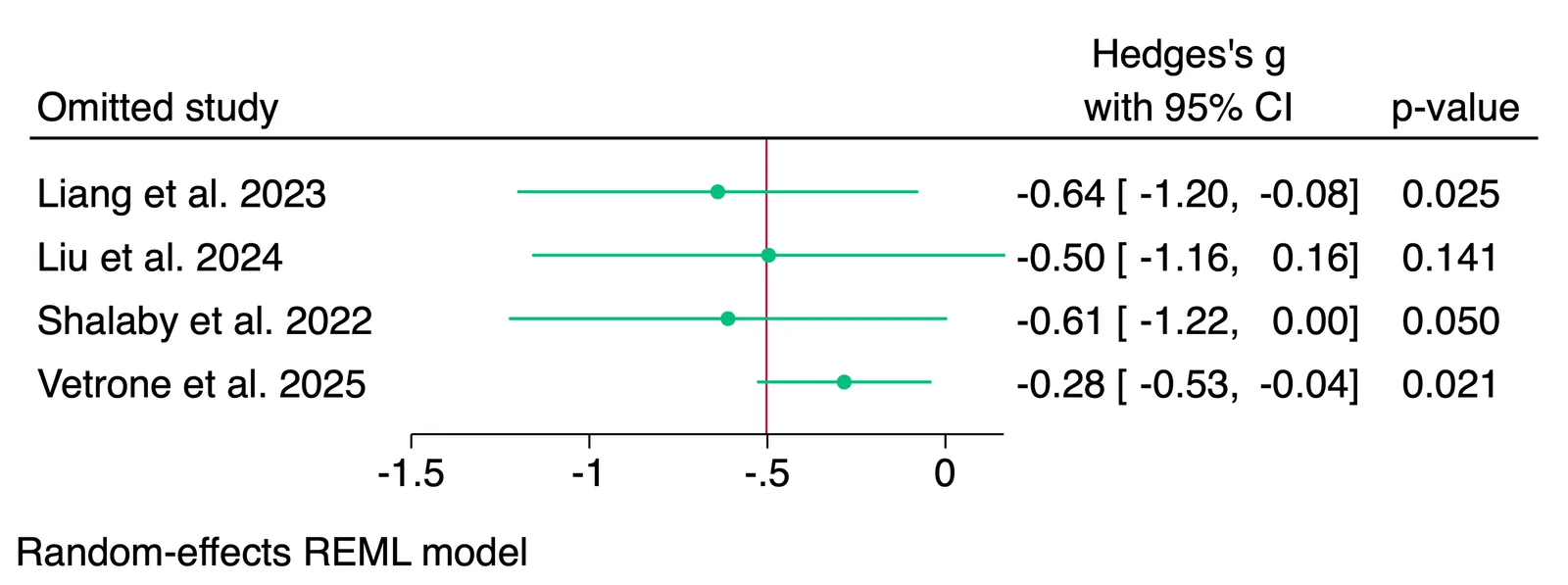
Leave-one-out sensitivity analysis of pain during movement after 6 hours. Studies: Liang et al. [14], Liu et al. [15], Shalaby et al. [16], Vetrone et al. [18]

**Figure 12:**
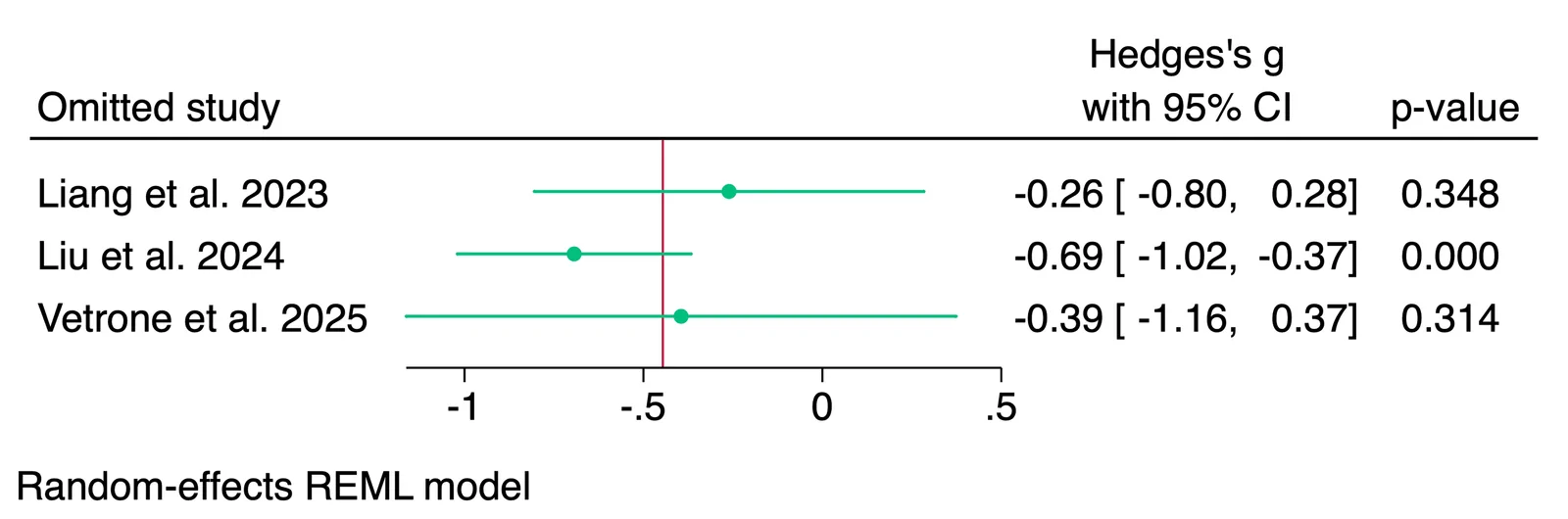
Leave-one-out sensitivity analysis of pain during movement after 48 hours. Studies: Liang et al. [14], Liu et al. [15], Vetrone et al. [18]

**Figure 13:**
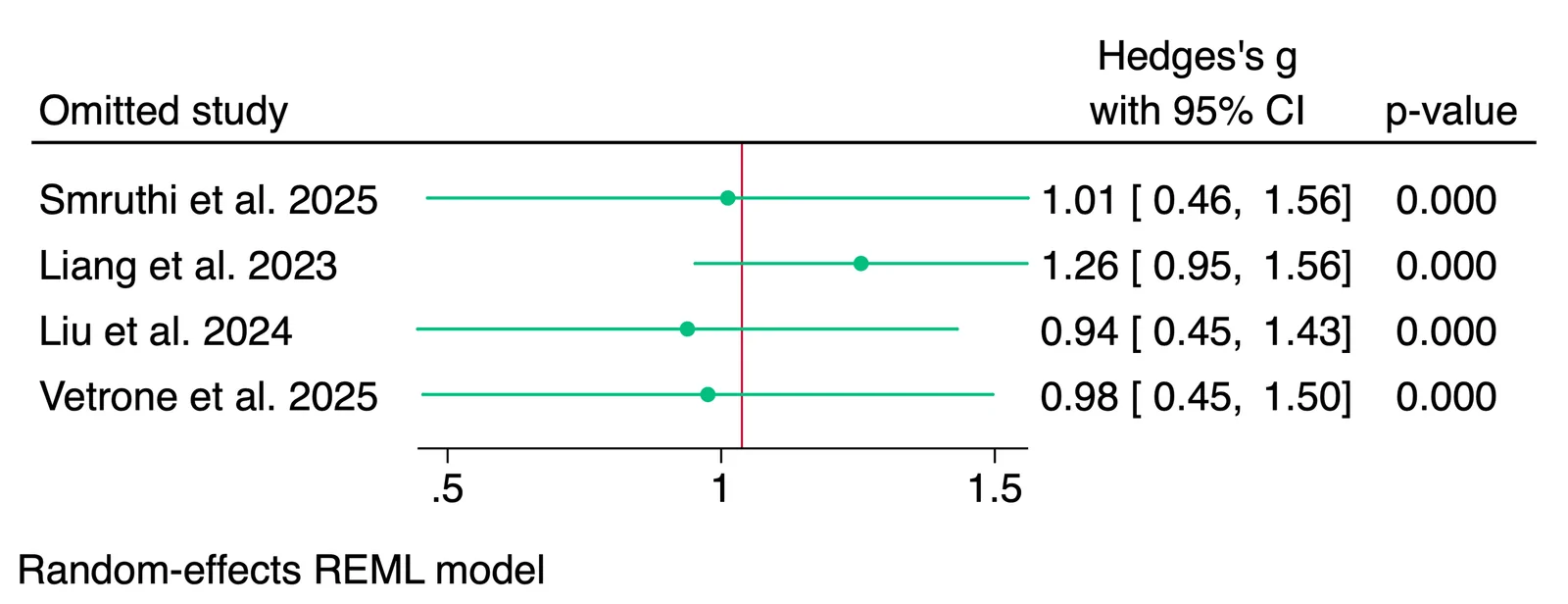
Leave-one-out sensitivity analysis of quadriceps muscle strength after 6 hours. Studies: Smruthi et al. [17], Liang et al. [14], Liu et al. [15], Vetrone et al. [18]

**Figure 14:**
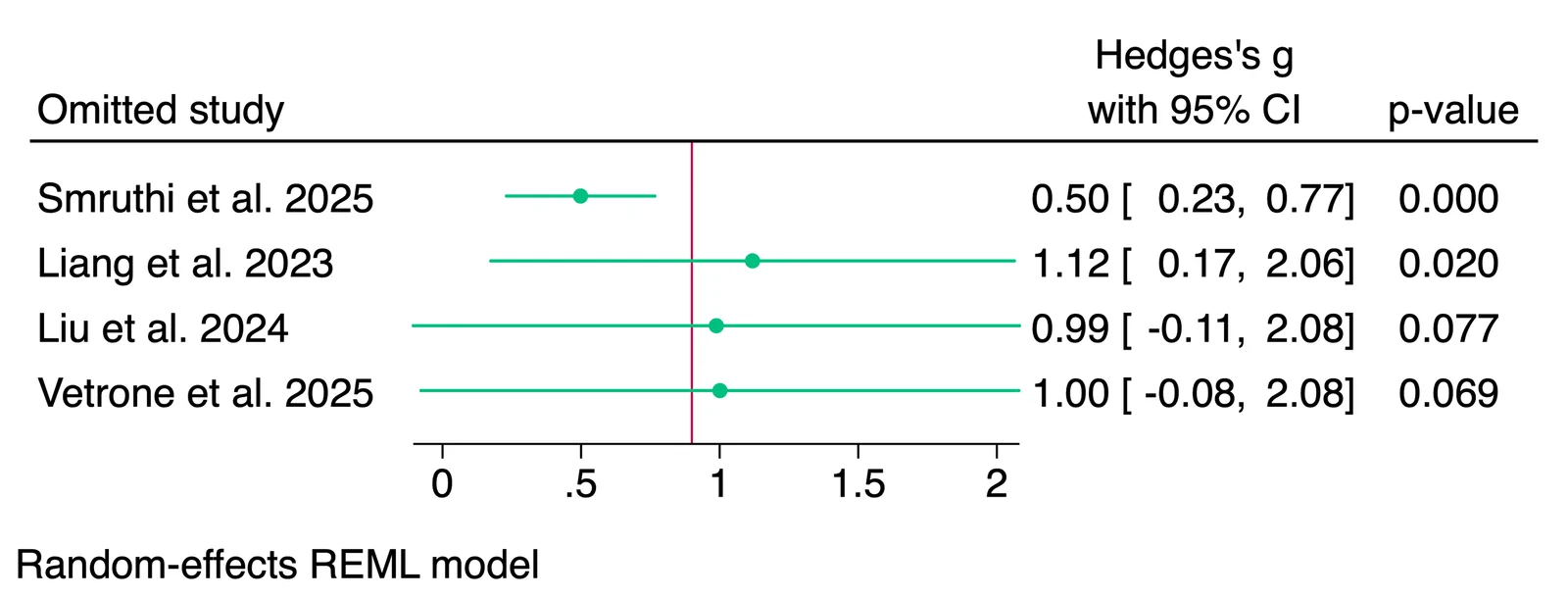
Leave-one-out sensitivity analysis of quadriceps muscle strength after 24 hours. Studies: Smruthi et al. [17], Liang et al. [14], Liu et al. [15], Vetrone et al. [18]

**Figure 15:**
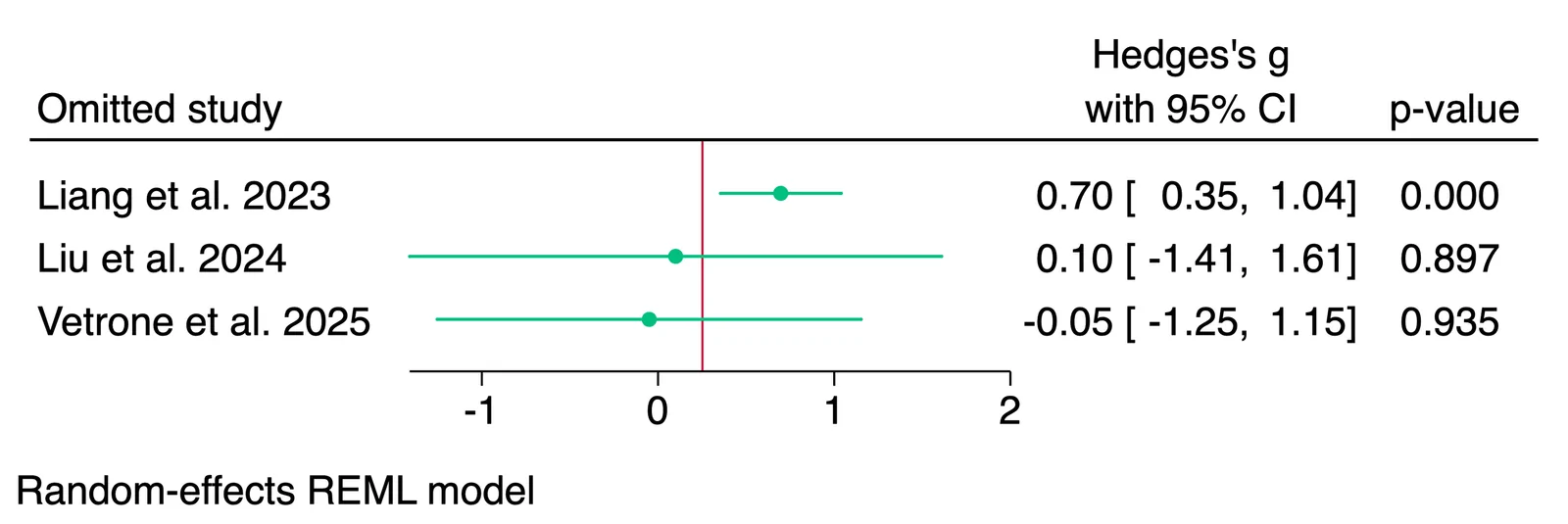
Leave-one-out sensitivity analysis of quadriceps muscle strength after 48 hours. Studies: Liang et al. [14], Liu et al. [15], Vetrone et al. [18]

**Figure 16:**
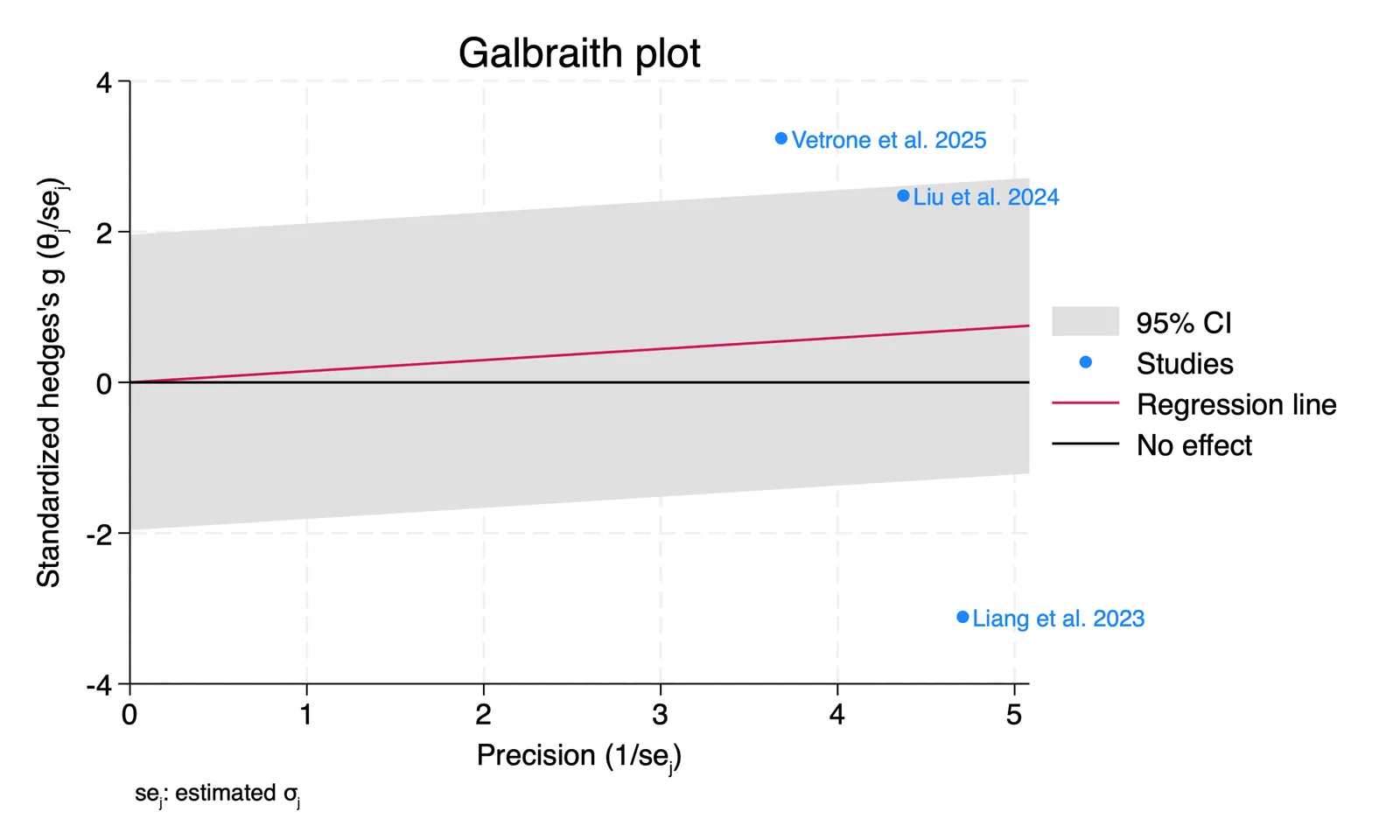
Galbraith plot of quadriceps muscle strength after 48 hours. Studies: Liang et al. [14], Liu et al. [15], Vetrone et al. [18]

**Figure 17:**
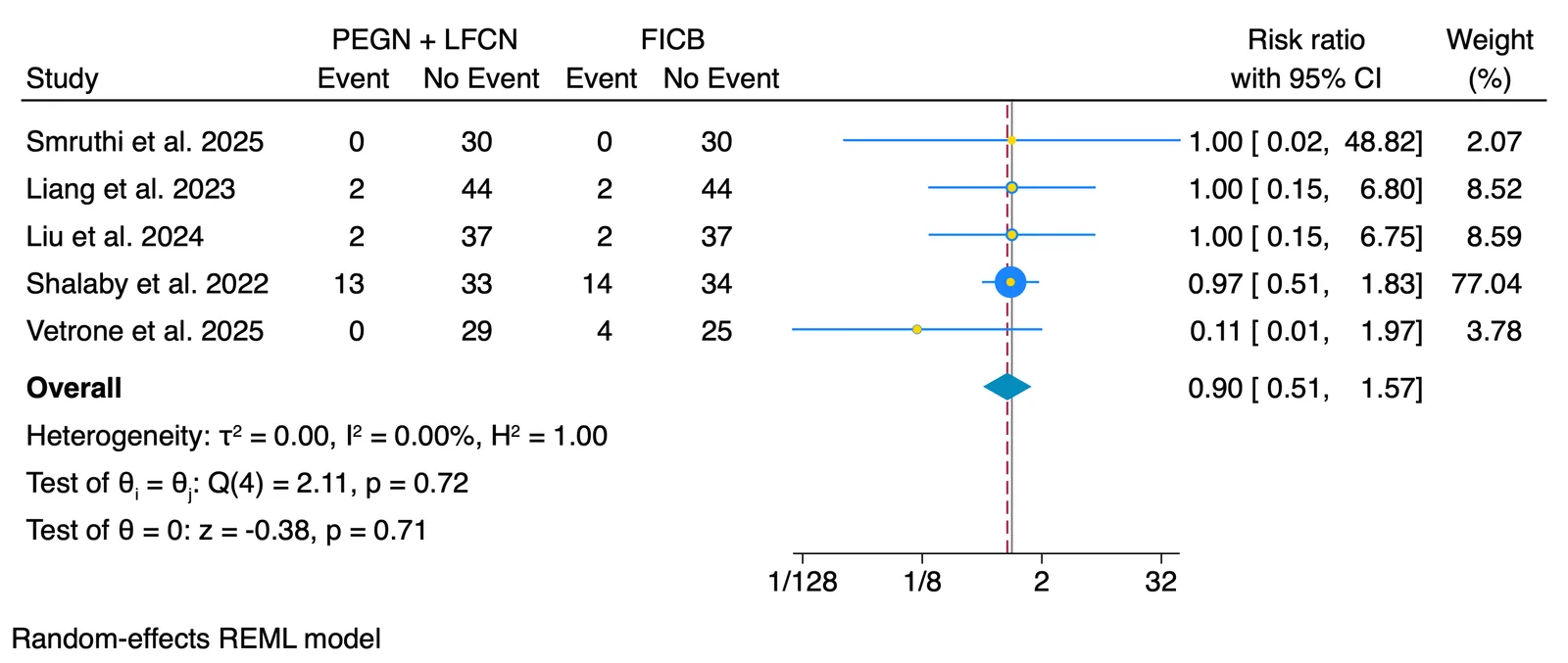
Forest plot of post-operative nausea and vomiting. Studies: Smruthi et al. [17], Liang et al. [14], Liu et al. [15], Shalaby et al. [16], Vetrone et al. [18]

**Figure 18:**
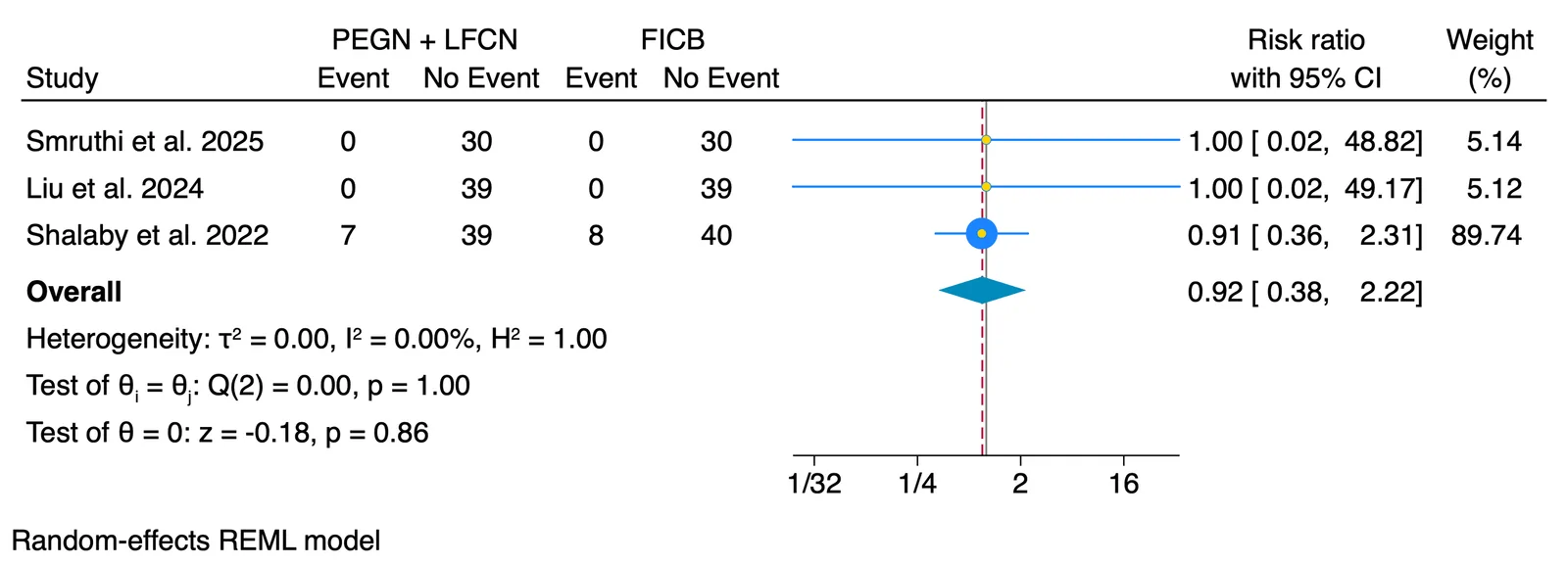
Forest plot of pruritus. Studies: Smruthi et al. [17], Liu et al. [15], Shalaby et al. [16]

**Figure 19:**
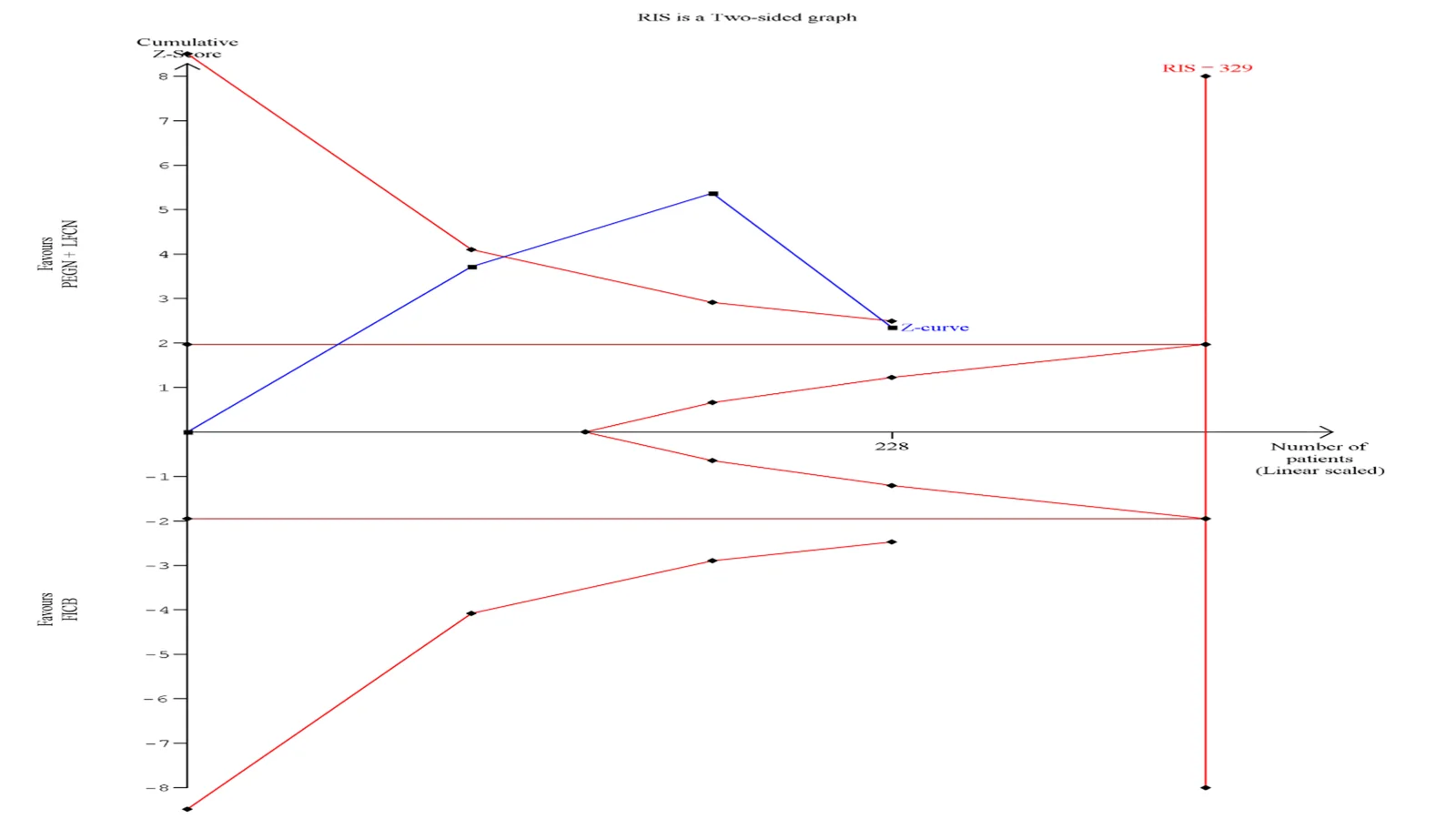
Trial sequential analysis of time to first ambulation.

**Figure 20:**
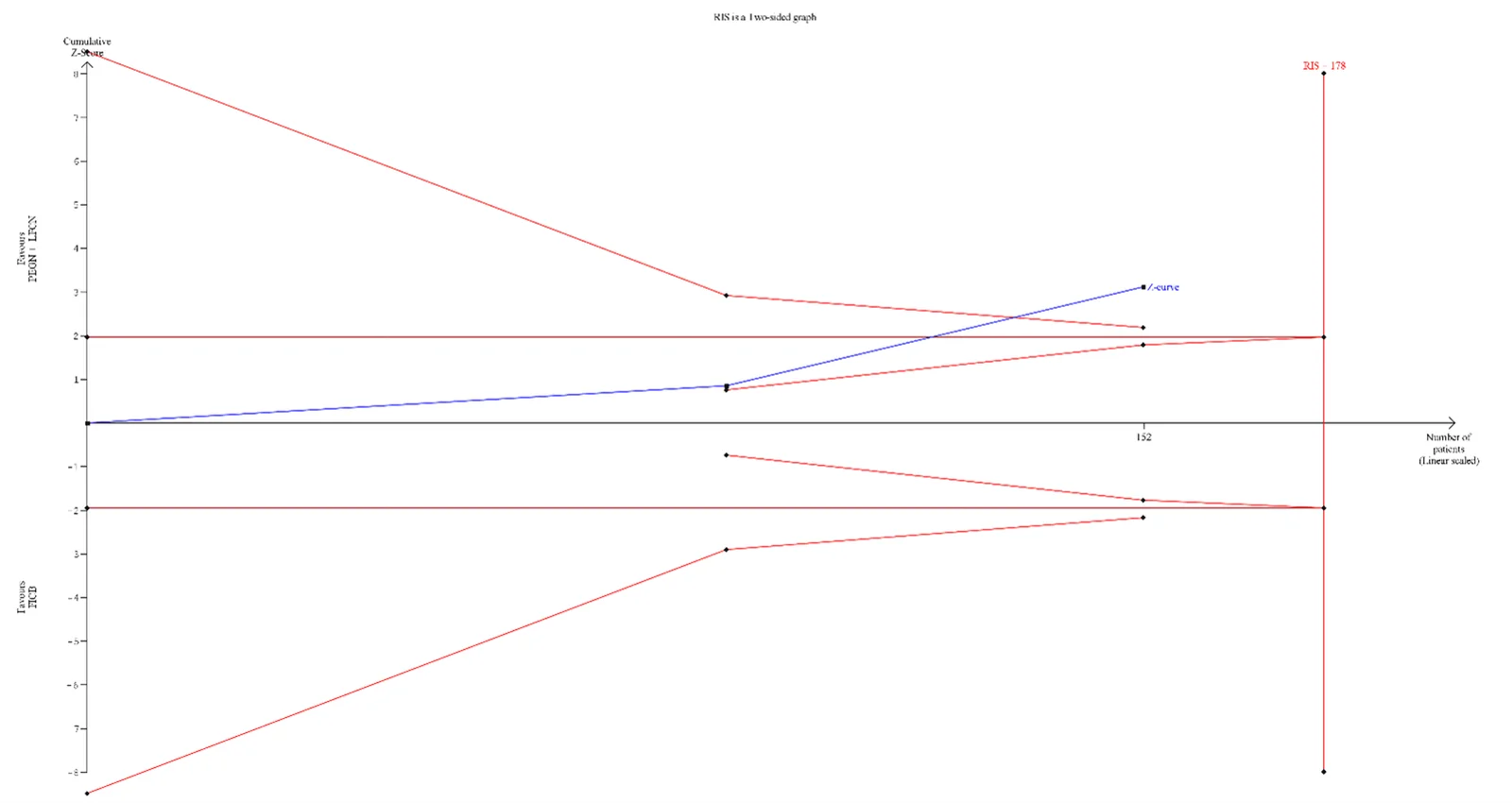
Trial sequential analysis of rescue analgesia.

## Appendices

Appendix A

Appendix B 

Appendix C 

Appendix D 

Appendix E 

Appendix F 

Appendix G 

Appendix H 

Appendix I 

Appendix J 

Appendix K 

Appendix L 

Appendix M 

Appendix N 
